# Complex formation between platelet-derived growth factor receptor β and transforming growth factor β receptor regulates the differentiation of mesenchymal stem cells into cancer-associated fibroblasts

**DOI:** 10.18632/oncotarget.26124

**Published:** 2018-09-25

**Authors:** Kaori Aoto, Kousei Ito, Shigeki Aoki

**Affiliations:** ^1^ Laboratory of Biopharmaceutics, Graduate School of Pharmaceutical Sciences, Chiba University, Inohana 1-8-1, Chuo-ku, Chiba-city, Chiba 260-8675, Japan

**Keywords:** cancer-associated fibroblast, PDGFRβ, TGFβ signaling, mesenchymal stem cell

## Abstract

Cancer-associated fibroblasts (CAFs) have recently gained attention as potent targets in cancer therapy because they are a crucial component of the tumor microenvironment and promote the growth and invasion of cancer cells. CAFs differentiate from fibroblasts, mesenchymal stem cells (MSCs), epithelial cells, and other cell types in response to transforming growth factor β (TGFβ) stimulation. The drugs tranilast, imatinib, and pirfenidone reportedly inhibit the differentiation of such cells into CAFs; however, it is unclear how they regulate TGFβ signaling. Here, we differentiated MSCs into CAFs *in vitro* and investigated which drugs suppressed this differentiation. Based on these results, we focused on platelet-derived growth factor (PDGF) receptor β (PDGFRβ) as a key molecule in the initiation of TGFβ signaling. PDGFRβ transmitted TGFβ signaling in MSCs by forming a complex with TGFβ receptor (TGFβR) independently of stimulation with its well-known ligand PDGF. Inhibitors of the differentiation of MSCs into CAFs attenuated complex formation between PDGFRβ and TGFβR. Moreover, PDGF stimulated PDGFRβ to a lesser extent in CAFs than in MSCs. This study indicates that PDGFRβ and TGFβ-TGFβR signaling cooperatively promote the differentiation of MSCs into CAFs in tumor microenvironments independently of canonical PDGF-PDGFR signaling. We propose that blockade of the interaction between PDGFRβ and TGFβR is a potential strategy to prevent TGFβ-mediated differentiation of MSCs into CAFs.

## INTRODUCTION

Tumor tissues are composed of cancer cells and the surrounding microenvironments. Tumor microenvironments contain vascular lymphatic networks, fibroblastic cells, inflammatory immune cells, mesenchymal stem cells (MSCs), and extracellular matrices [1, 2]. Much data acquired *in vitro* and *in vivo* suggest that fibroblastic cells are important for cancer progression, in addition to the tumor stroma [3]. In particular, cancer-associated fibroblasts (CAFs), a subpopulation of fibroblastic cells, promote tumor growth and progression [4]. CAFs accelerate remodeling of the extracellular matrix, angiogenesis, and recruitment of inflammatory immune cells, and also secrete growth factors that increase the proliferation of cancer cells [5, 6]. In addition, CAFs have important functions in tumor microenvironments; therefore, effective anti-cancer therapies that target CAFs in addition to cancer cells must be developed.

CAFs differentiate from fibroblasts, MSCs, epithelial cells, endothelial cells, and other cell types in tumor microenvironments [7]. Expression of α-smooth muscle actin (αSMA), N-cadherin, fibroblast surface protein, fibroblast activation protein, and vimentin is much higher in CAFs than in normal fibroblasts [8]; however, there is no known specific marker protein of CAFs. Transforming growth factor β (TGFβ) is a cytokine mainly released from cancer cells that plays an important role in differentiation into CAFs [9, 10]. Smad proteins are phosphorylated upon activation of TGFβ receptor (TGFβR) by its specific ligand TGFβ [11]. Phosphorylated Smad proteins subsequently translocate into the nucleus and function as transcription factors to induce expression of CAF-related genes, including αSMA [11].

There are two isoforms of platelet-derived growth factor (PDGF) receptors (PDGFRs): PDGFRα and PDGFRβ [12]. PDGFRs control the functions of MSCs via remodeling the actin cytoskeleton, inducing cell migration by activating phosphoinositide 3-kinase (PI3K) and phospholipase C-γ, and promoting cell growth by activating the mitogen-activated protein kinase (MAPK) pathway and Src [13]. Furthermore, PDGFRs stimulate the growth of squamous cell carcinomas, such as renal cell, ovarian, and prostate cancers [14], promote vascularization by recruiting pericytes to blood vessels within tumor tissues [15], and facilitate lymphatic regeneration in fibrosarcomas [16]. All these effects exacerbate tumor pathology. In addition, PDGFR expression in fibroblastic cells within the microenvironment of breast cancer is positively correlated with the pathological grade, HER2 expression, and shortening of progression-free survival [17]. Stimulation with PDGF, the ligand of PDGFRs, is required for recruitment of fibroblastic cells to tumor tissues [13]. In summary, PDGFRs promote cancer malignancy via various intracellular mechanisms.

Although a clinical strategy that directly suppresses the functions of CAFs has not been approved, several pharmaceuticals that are already clinically used have been suggested to inhibit fibroblast activation, differentiation into CAFs, and growth of CAFs in the laboratory. Among these pharmaceuticals, tranilast, which inhibits release of chemical mediators such as histamines from mast cells and is clinically used as an anti-allergy medication [18], suppresses the growth of CAFs and their secretion of TGFβ in mice bearing lymphosarcomas or Lewis lung carcinomas [19]. Imatinib, a tyrosine kinase inhibitor (TKI) that binds to c-ABL, BCR-ABL, and c-KIT and is clinically used to treat chronic myeloid leukemia and gastrointestinal stromal tumors [20], prevents fibrosis in a mouse model of bleomycin-induced lung fibrosis [21] and reduces αSMA expression in CAFs derived from colon metastatic lesions of patients [22]. Pirfenidone, which inhibits the growth and activation of fibroblasts and is clinically used to treat idiopathic pulmonary fibrosis [23], suppresses the differentiation of human pulmonary fibroblasts into myofibroblasts upon exposure to TGFβ [24]. Although these drugs have been suggested to inhibit differentiation into CAFs by reducing production of TGFβ and phosphorylation of Smad proteins downstream of TGFβ signaling [19, 24], it is unclear how they regulate TGFβ signaling.

Inhibition of the differentiation of normal cells, including MSCs, into CAFs within tumor microenvironments is a potential anti-cancer strategy. Therefore, we sought to elucidate the detailed molecular mechanism by which TGFβ stimulation induces the differentiation of these cells into CAFs and how the aforementioned drugs capable of suppressing this differentiation interfere with TGFβ signaling. Here, we differentiated MSCs into CAFs via TGFβ stimulation *in vitro* and investigated how this differentiation was suppressed by various drugs. We demonstrated that PDGFRβ and TGFβ signaling cooperatively promote the differentiation of MSCs into CAFs upon TGFβ exposure independently of PDGF stimulation and that complex formation between PDGFRβ and TGFβR is important for this differentiation.

## RESULTS

### MSCs differentiate into CAFs upon TGFβ stimulation

**Figure 1:**
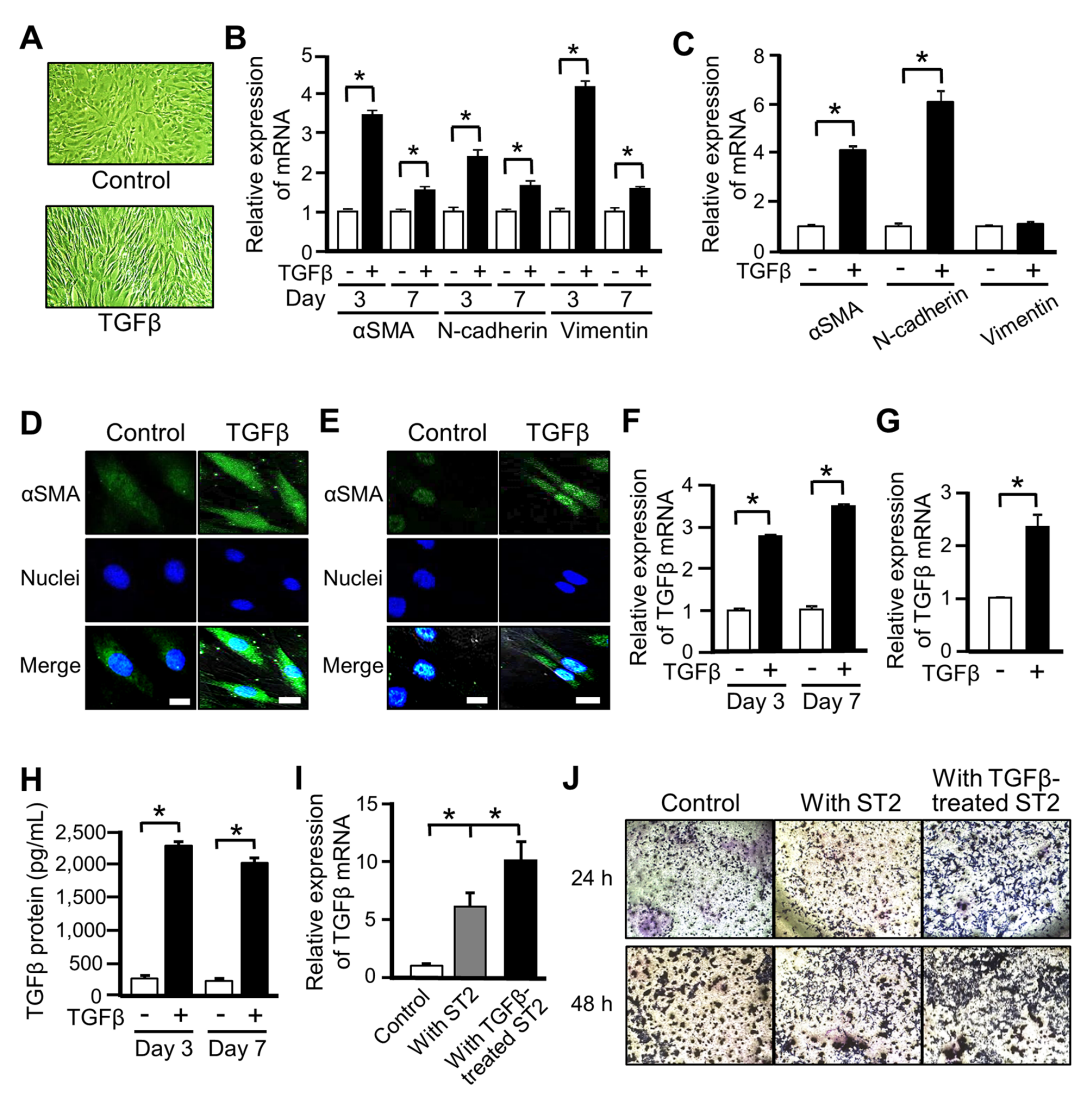
Mesenchymal stem cells (MSCs) differentiate into cancer-associated fibroblasts (CAFs) upon TGFβ stimulation **(A)** Microscopic images of ST2 cells after stimulation with TGFβ (10 ng/mL) for 48 h. Magnification ×100. **(B, C)** Quantitative RT-PCR analysis was performed to measure mRNA expression of the CAF markers αSMA, N-cadherin, and vimentin in ST2 cells treated with TGFβ for 3 and 7 days (B) and in human MSCs treated with TGFβ for 3 days (C). Each bar represents the mean ± SD (N=3), ^*^P < 0.05. **(D, E)** Immunostaining of αSMA (green) was performed in ST2 cells (D) and human MSCs (E) treated with TGFβ for 48 h. Each scale bar indicates 20 μm. Nuclei were stained with TO-PRO-3 (blue). **(F, G)** Quantitative RT-PCR analysis was performed to measure mRNA expression of TGFβ in ST2 cells treated with TGFβ for 3 and 7 days (F) and in human MSCs treated with TGFβ for 3 days (G). Each bar represents the mean ± SD (N=3), ^*^P < 0.05. **(H)** Secretion of TGFβ by ST2 cells treated with TGFβ for 3 and 7 days was assessed by an ELISA. Each bar represents the mean ± SE (N=3), ^*^P < 0.05. **(I)** B16 cells were co-cultured with non-treated or TGFβ-treated ST2 cells in a transwell plate for 7 days, and mRNA expression of TGFβ in B16 cells was measured by quantitative RT-PCR analysis. Each bar represents the mean ± SD (N=3), ^*^P < 0.05. **(J)** B16 cells were co-cultured with non-treated or TGFβ-treated ST2 cells for 24 or 48 h, and invaded B16 cells were stained with crystal violet (deep blue).

Among the various cell types that reportedly give rise to CAFs, we focused on MSCs, which can differentiate into various cell types including osteoblastic cells, adipocytes, muscle cells, and CAFs [25]. We first investigated the differentiation of murine MSC-like ST2 cells into CAFs upon exposure to TGFβ. ST2 cells became spindle-shaped following TGFβ stimulation (Figure 1A). mRNA expression of αSMA, N-cadherin, and vimentin, which are markers of differentiation into CAFs, was significantly increased in TGFβ-treated ST2 cells, and these increases were sustained for 1 week (Figure 1B). Similarly, mRNA expression of αSMA and N-cadherin was increased in human primary MSCs treated with TGFβ (Figure 1C). Moreover, the intracellular level of αSMA protein was increased in ST2 cells and human MSCs exposed to TGFβ (Figures 1D and 1E). These results confirm that ST2 cells and human MSCs differentiated into CAF-like cells upon TGFβ stimulation.

CAFs reportedly also release TGFβ and increase the invasive ability of cancer cells in tumor microenvironments [7]. mRNA expression (Figures 1F and 1G) and secretion (Figure 1H) of TGFβ were increased in TGFβ-stimulated ST2 cells (Figures 1F and 1H) and human MSCs (Figure 1G). Next, we evaluated the effect of CAFs on the invasive ability of cancer cells. Upon co-culture in a transwell plate, mRNA expression of TGFβ was higher in mouse melanoma B16 cells co-cultured with TGFβ-stimulated ST2 cells than in those co-cultured with non-stimulated ST2 cells (Figure 1I). Moreover, the invasive ability of the former cells was higher than that of the latter cells (Figure 1J). These results indicate that ST2 cells differentiate into CAFs upon TGFβ treatment and that these CAFs increase the malignancy of cancer cells.

### PDGFRs may be involved in the suppressive effects of various drugs on differentiation into CAFs

**Figure 2:**
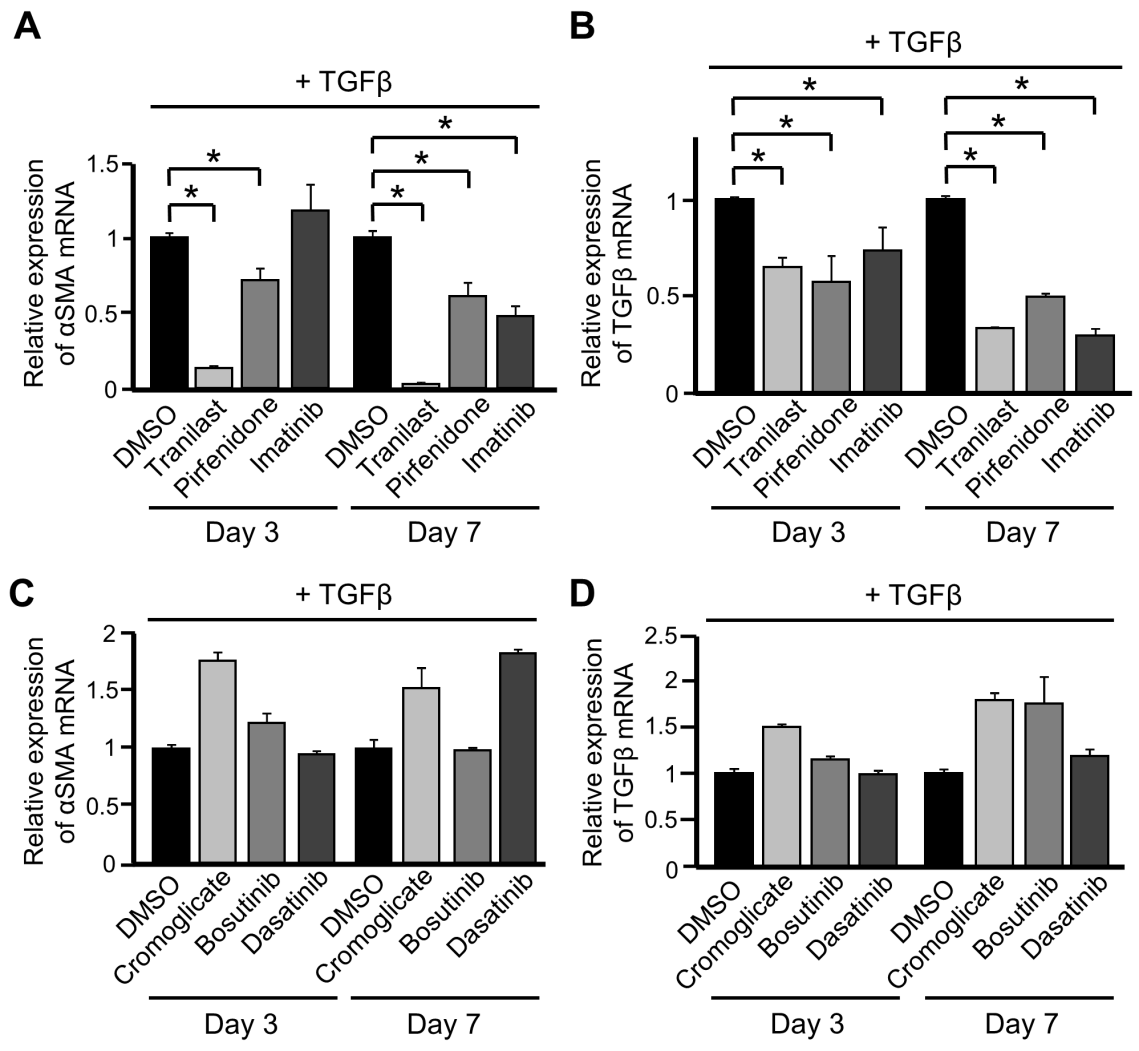
PDGFRs may be involved in the suppressive effects of various drugs on differentiation into cancer-associated fibroblasts (CAFs) **(A, B)** ST2 cells were pre-treated with tranilast (100 μM), pirfenidone (0.2 mg/mL), or imatinib (10 μM) for 1 h and then stimulated with TGFβ (10 ng/mL) for 3 and 7 days. Quantitative RT-PCR analysis was performed to measure mRNA expression of αSMA (A) and TGFβ (B). Each bar represents the mean ± SD (N=3), ^*^P < 0.05. **(C, D)** ST2 cells were pre-treated with cromoglicate (100 μM), bosutinib (100 nM), or dasatinib (10 nM) for 1 h and then stimulated with TGFβ for 3 and 7 days. Quantitative RT-PCR analysis was performed to measure mRNA expression of αSMA (C) and TGFβ (D). Each bar represents the mean ± SD (N=3).

Tranilast, pirfenidone, and imatinib are reported to suppress differentiation into CAFs, as described in the Introduction. We investigated whether treatment with these drugs inhibited the increase in expression of CAF markers upon TGFβ stimulation in our experimental conditions. Exposure to any of these three drugs suppressed the increases in mRNA expression of αSMA and TGFβ in TGFβ-treated ST2 cells (Figures 2A and 2B).

Next, we evaluated whether cromoglicate, another inhibitor of chemical mediator release, and dasatinib and bosutinib, which are TKIs, also inhibited the differentiation of ST2 cells into CAFs. Exposure to these three drugs did not suppress the increases in mRNA expression of αSMA and TGFβ in TGFβ-stimulated ST2 cells (Figures 2C and 2D). These results suggest that the main pharmacological actions of tranilast and imatinib are not directly involved in the suppression of differentiation into CAFs.

Imatinib and these two second-generation TKIs (dasatinib and bosutinib) target different kinases. Puttini et al. investigated the suppressive effects of bosutinib and imatinib on growth of leukemia cells expressing Bcr-Abl, Ba/F3 cells expressing PDGFRβ, and gastrointestinal stromal tumor cells expressing c-KIT, and calculated their IC_50_ values [26]. They estimated that the IC_50_ values of imatinib and bosutinib in Ba/F3 cells expressing PDGFRβ are 3.4 and 370 nmol/L, respectively, suggesting that imatinib strongly inhibits PDGFRβ [26]. Therefore, we hypothesized that PDGFRs, including PDGFRβ, induce differentiation into CAFs in cooperation with TGFβ signaling.

### PDGFRβ is involved in the differentiation of MSCs into CAFs

**Figure 3:**
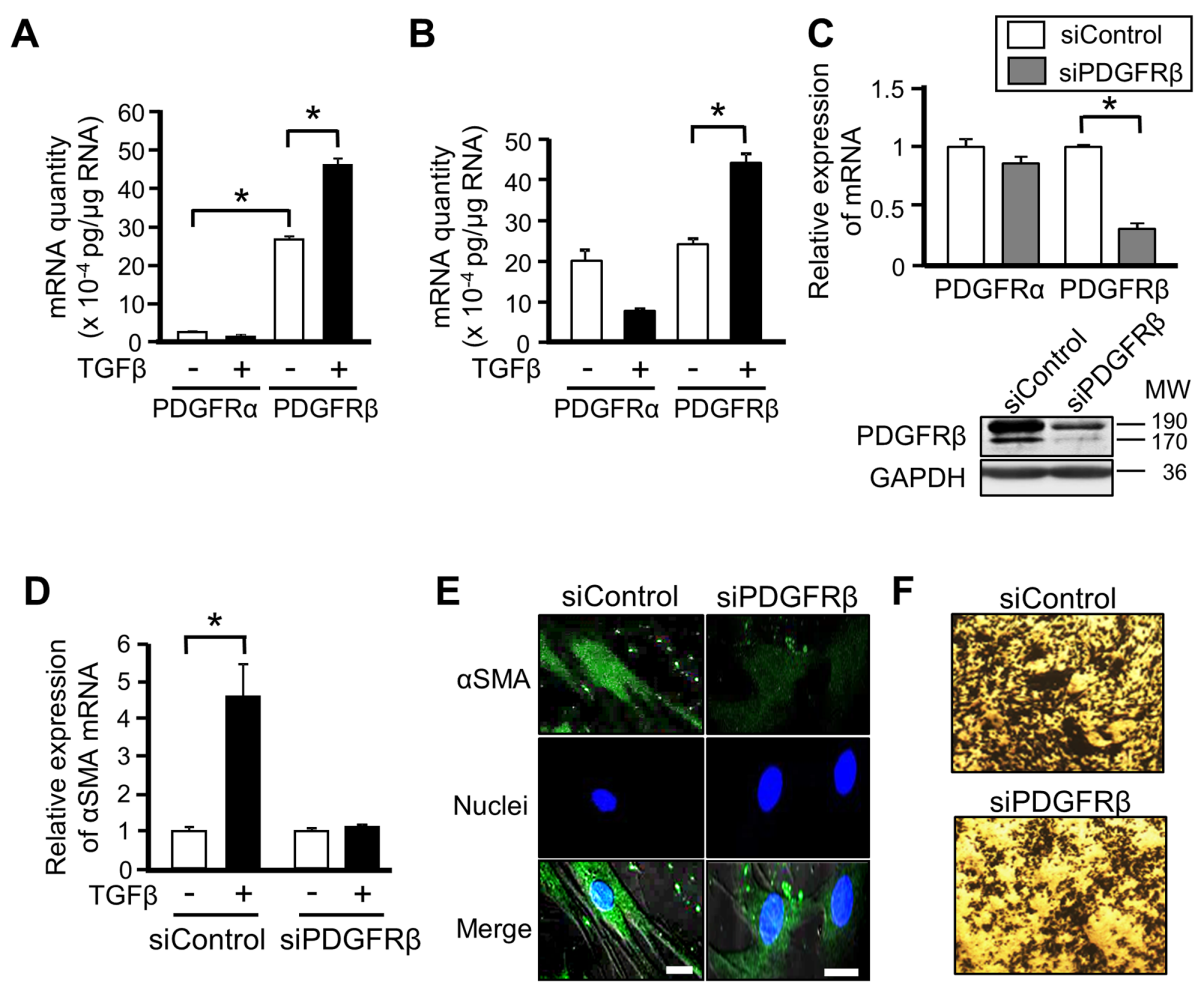
PDGFRβ is involved in the differentiation of mesenchymal stem cells (MSCs) into cancer-associated fibroblasts **(A, B)** Absolute quantitative RT-PCR analyses were performed to measure mRNA expression of PDGFRα and PDGFRβ in ST2 cells (A) and human MSCs (B) stimulated with or without TGFβ (10 ng/mL) for 48 h. The absolute quantity of mRNA was converted to picograms. Each bar represents the mean ± SD (N=3), ^*^P < 0.05. **(C)** ST2 cells were transfected with PDGFRβ-targeting or negative control siRNA for 48 h, and then mRNA expression of PDGFRα and PDGFRβ was measured by quantitative RT-PCR analysis. Each bar represents the mean ± SD (N=3), ^*^P < 0.05. Lysates of ST2 cells transfected with the indicated siRNA were subjected to western blotting with anti-PDGFRβ and anti-GAPDH antibodies. **(D)** ST2 cells were transfected with PDGFRβ-targeting or negative control siRNA for 48 h and then treated with TGFβ for 48 h. Quantitative RT-PCR analysis was performed to measure mRNA expression of αSMA. Each bar represents the mean ± SD (N=3), ^*^P < 0.05. **(E)** ST2 cells described in (D) were immunostained for αSMA. Each scale bar indicates 20 μm. Nuclei were stained with TO-PRO-3 (blue). **(F)** ST2 cells were transfected with PDGFRβ-targeting or negative control siRNA for 48 h, treated with TGFβ for 24 h, and co-cultured with B16 cells for 48 h. Invaded B16 cells were stained with crystal violet (deep blue).

There are two subtypes of PDGFRs: PDGFRα and PDGFRβ [12]. We quantified the absolute expression levels of these two PDGFR subtypes in ST2 cells and human MSCs, and evaluated the changes in their expression upon TGFβ stimulation. mRNA expression of PDGFRβ was higher than that of PDGFRα in ST2 cells, and TGFβ stimulation significantly increased mRNA expression of PDGFRβ, but decreased that of PDGFRα (Figure 3A). Although basal mRNA expression of PDGFRα and PDGFRβ did not differ in human MSCs, TGFβ stimulation significantly increased mRNA expression of PDGFRβ, similar to its effect in ST2 cells (Figure 3B). These results suggest that PDGFRβ is involved in the differentiation of MSCs into CAFs.

We further evaluated the involvement of PDGFRβ in the differentiation of MSCs into CAFs by depleting its expression. Transfection of PDGFRβ-targeting siRNA markedly reduced mRNA and protein expression of PDGFRβ in ST2 cells (Figure 3C). The increases in mRNA and protein expression of αSMA upon TGFβ stimulation were abrogated in PDGFRβ-knockdown ST2 cells (Figures 3D and 3E). In addition, knockdown of PDGFRβ attenuated the increase in the invasive ability of B16 cells upon co-culture with TGFβ-treated ST2 cells (Figure 3F). These results indicate that PDGFRβ and TGFβ signaling cooperatively promote the differentiation of MSCs into CAFs.

### PDGF-PDGFRβ signaling does not affect the differentiation of MSCs into CAFs

**Figure 4:**
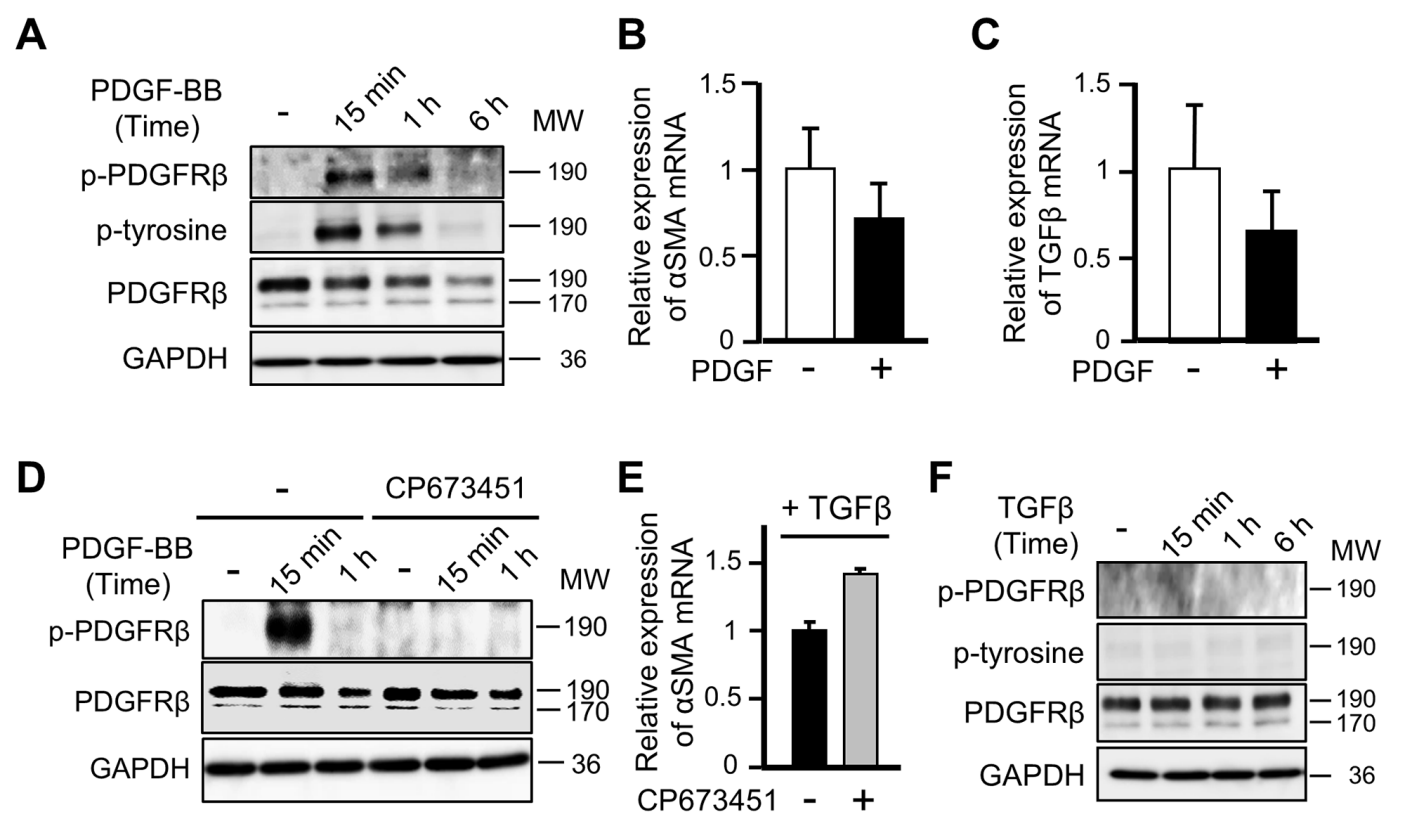
PDGF-PDGFRβ signaling does not affect the differentiation of mesenchymal stem cells into cancer-associated fibroblasts **(A)** Lysates of ST2 cells treated with PDGF-BB (20 ng/mL) for 15 min, 1 h, or 6 h were subjected to western blotting with anti-p-PDGFRβ, anti-p-tyrosine, anti-PDGFRβ, and anti-GAPDH antibodies. **(B, C)** Quantitative RT-PCR analyses were performed to measure mRNA expression of αSMA (B) and TGFβ (C) in ST2 cells treated with PDGF-BB for 48 h. Each bar represents the mean ± SD (N=3). **(D)** ST2 cells were pre-treated with CP673451 (50 nM) for 2 h and then stimulated with PDGF-BB for 15 min or 1 h. Cell lysates were subjected to western blotting with anti-p-PDGFRβ, anti-PDGFRβ, and anti-GAPDH antibodies. **(E)** ST2 cells were pre-treated with CP673451 for 1 h and then stimulated with TGFβ (10 ng/mL) for 3 days. Quantitative RT-PCR analysis was performed to measure mRNA expression of αSMA. Each bar represents the mean ± SD (N=3). **(F)** Lysates of ST2 cells stimulated with TGFβ for 15 min, 1 h, or 6 h were subjected to western blotting with anti-p-PDGFRβ, anti-p-tyrosine, anti-PDGFRβ, and anti-GAPDH antibodies.

PDGFRβ is autophosphorylated in response to its well-known ligand PDGF-BB, leading to activation of downstream signaling molecules in the PI3K and MAPK pathways [27]. Therefore, we evaluated whether PDGF stimulation regulates the differentiation of ST2 cells into CAFs by inducing phosphorylation of PDGFRβ. Although PDGF-BB stimulation induced phosphorylation of PDGFRβ (Figure 4A) and promoted the proliferation of ST2 cells (data not shown), it decreased, rather than increased, mRNA expression of αSMA and TGFβ (Figures 4B and 4C). Moreover, exposure to CP673451, which inhibits phosphorylation of PDGFRβ, enhanced, rather than suppressed, the increase in mRNA expression of αSMA in TGFβ-treated ST2 cells (Figures 4D and 4E). In addition, TGFβ stimulation, which is necessary for differentiation into CAFs, did not induce phosphorylation of PDGFRβ in ST2 cells (Figure 4F). These results indicate that PDGFRβ is not phosphorylated during the differentiation of MSCs into CAFs upon TGFβ stimulation and that this differentiation occurs independently of the classical PDGF-PDGFR signaling cascade.

### PDGFRβ induces the differentiation of MSCs into CAFs by interacting with TGFβR

**Figure 5:**
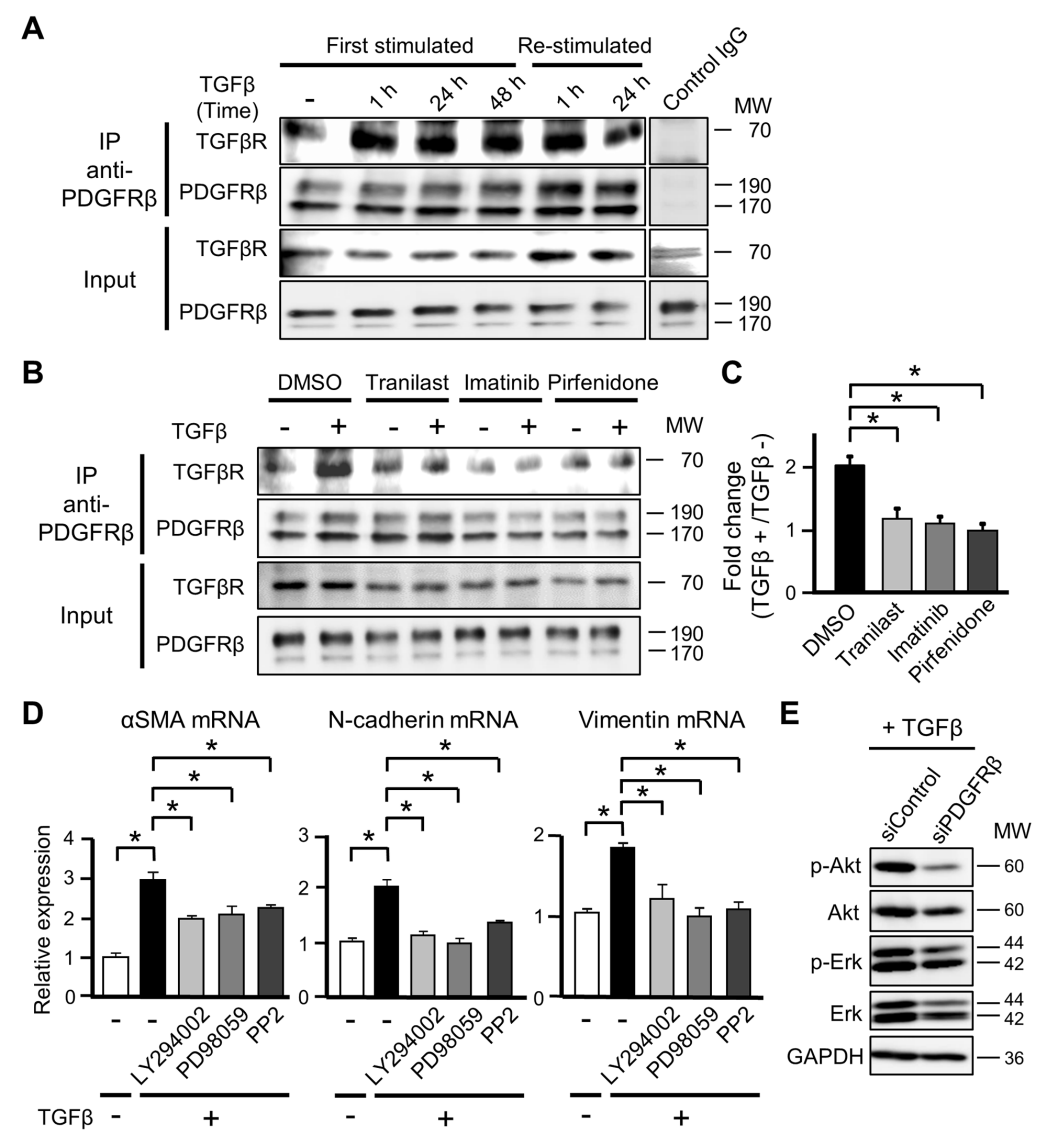
PDGFRβ induces the differentiation of mesenchymal stem cells into cancer-associated fibroblasts by interacting with TGFβ receptor (TGFβR) **(A)** ST2 cells were stimulated with TGFβ (10 ng/mL) for 1, 24, or 48 h (First stimulated), and ST2 cells stimulated with TGFβ for 48 h were re-stimulated with TGFβ for 1 or 24 h (Re-stimulated). Cell lysates were subjected to immunoprecipitation (IP) using an anti-PDGFRβ antibody or IgG isotype control. Immunoprecipitates were subjected to western blotting with anti-TGFβR and anti-PDGFRβ antibodies. Total cell lysates were examined in parallel. **(B)** ST2 cells were pre-treated with tranilast (100 μM), imatinib (10 μM), or pirfenidone (0.2 mg/mL) for 12 h and then stimulated with TGFβ for 1 h. Cell lysates were subjected to immunoprecipitation using an anti-PDGFRβ antibody. Immunoprecipitates were subjected to western blotting with anti-TGFβR and anti-PDGFRβ antibodies. Total cell lysates were examined in parallel. **(C)** The intensities of the bands in (B) were quantified, and the intensity of the TGFβR band relative to that of the PDGFRβ band was determined. Fold changes in the intensity of the TGFβR band were determined by comparing the samples obtained before and after TGFβ stimulation. Each bar represents the mean ± SD (N=3), ^*^P < 0.05. **(D)** ST2 cells were pre-treated with LY294002 (3 μM), PD98059 (20 μM), or PP2 (500 nM) for 1 h and then stimulated with TGFβ for 48 h. Quantitative RT-PCR analyses were performed to measure mRNA expression of αSMA, N-cadherin, and vimentin. Each bar represents the mean ± SD (N=3), ^*^P < 0.05. **(E)** ST2 cells were transfected with PDGFRβ-targeting or negative control siRNA for 48 h and then treated with TGFβ for 24 h. Cell lysates were subjected to western blotting with anti-p-Akt, anti-Akt, anti-p-Erk, anti-Erk, and anti-GAPDH antibodies.

TGFβ transmits intracellular signals by binding to the heterotetramer TGFβR, which is composed of two type I and two type II receptors [28]. Given that inhibition of PDGFRβ phosphorylation did not perturb the differentiation of MSCs into CAFs, we investigated whether PDGFRβ transmits TGFβ-TGFβR signaling by interacting with TGFβR. Immunoprecipitation analyses using an anti-PDGFRβ antibody indicated that complex formation between PDGFRβ and TGFβR was higher at 1 h after TGFβ stimulation than prior to stimulation and that this effect was sustained at 48 h after stimulation (Figure 5A). In addition, no further complex formation between PDGFRβ and TGFβR was observed when CAFs that had differentiated from ST2 cells in response to TGFβ were re-stimulated with TGFβ (Figure 5A). These results suggest that the interaction between PDGFRβ and TGFβR is important for initiation of the differentiation of MSCs into CAFs and that this complex is maintained in CAFs.

We evaluated whether exposure to tranilast, imatinib, and pirfenidone, which suppress differentiation into CAFs, decreased complex formation between PDGFRβ and TGFβR in ST2 cells. The increase in complex formation between PDGFRβ and TGFβR was attenuated in ST2 cells pre-treated with each drug for 12 h and then exposed to TGFβ for 1 h (Figures 5B and 5C). This finding suggests that these drugs suppress the differentiation of MSCs into CAFs by physicochemically preventing the interaction between PDGFRβ and TGFβR.

### PDGFRβ contributes to the activation of non-canonical TGFβ signaling

Binding of TGFβ to its receptor promotes phosphorylation of the transcription factors Smad2/3, and then phosphorylated Smad2/3 translocate to the nucleus and induce expression of their target genes, including αSMA [28]. We evaluated whether PDGFRβ affects activation of Smad2/3 downstream of TGFβ signaling. The level of Smad2/3 phosphorylation did not significantly differ between control and PDGFRβ-knockdown ST2 cells following TGFβ exposure (data not shown), indicating that PDGFRβ does not directly affect activation of Smad2/3 upon TGFβ stimulation.

In addition to the canonical Smad-dependent TGFβ signaling pathway, TGFβ also activates Smad-independent non-canonical pathways. These pathways are regulated by various molecules, including Erk, JNK/p38, Rho-like GTPases, PI3K/Akt, and Src [29]. Differentiation into CAFs is suppressed by reducing the activities of non-canonical pathways, even if the canonical TGFβ signaling pathway is active [30]. Exposure to TGFβ plus a non-canonical pathway inhibitor (the PI3K inhibitor LY294002, the MEK inhibitor PD98059, or the Src inhibitor PP2) significantly suppressed the increases in mRNA expression of αSMA, N-cadherin, and vimentin in ST2 cells (Figure 5D). In addition, knockdown of PDGFRβ attenuated phosphorylation of Akt and Erk in TGFβ-treated ST2 cells (Figure 5E). Consequently, we speculate that PDGFRβ is necessary to activate non-canonical TGFβ signaling, which contributes to the differentiation of MSCs into CAFs.

### TGFβ stimulation alters the functions of PDGFRβ in MSCs

**Figure 6:**
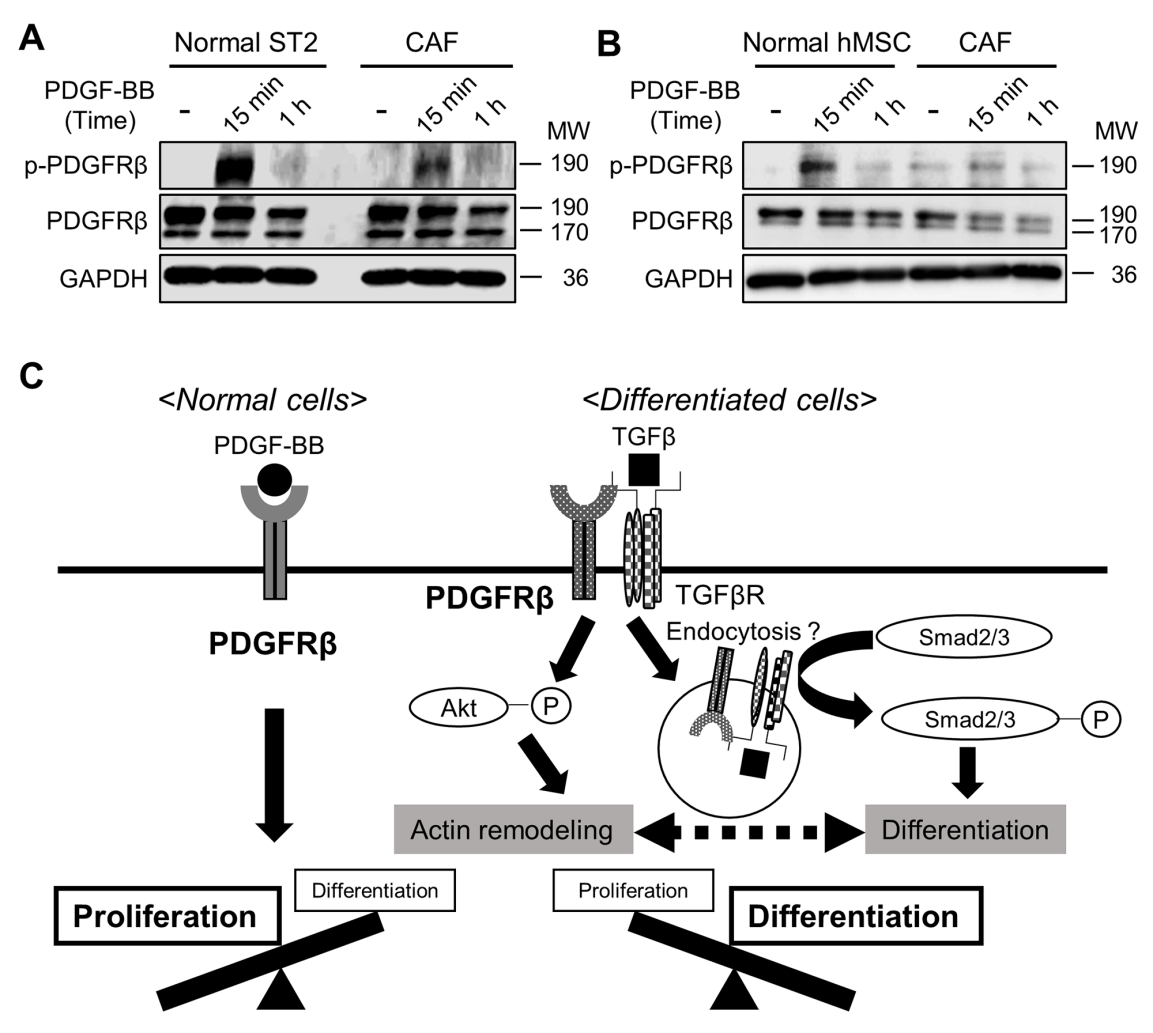
Stimulation with TGFβ alters the functions of PDGFRβ in mesenchymal stem cells (MSCs) **(A, B)** ST2 cells (A) and human MSCs (B) were pre-treated with or without TGFβ (10 ng/mL) for 72 h and then stimulated with PDGF-BB (20 ng/mL) for 15 min or 1 h. Cell lysates were subjected to western blotting with anti-p-PDGFRβ, anti-PDGFRβ, and anti-GAPDH antibodies. **(C)** A proposed scheme of how the functions of PDGFRβ change during differentiation into cancer-associated fibroblasts.

PDGFRs reportedly regulate both cell proliferation and migration/differentiation [27]. We hypothesized that after MSCs have differentiated into CAFs, PDGFRβ helps to maintain CAFs via TGFβ signaling, rather than modulating cell proliferation in response to PDGF. To investigate this, we compared the level of PDGFRβ phosphorylation in response to PDGF-BB stimulation between non-stimulated MSCs and MSCs that had been stimulated with TGFβ for 72 h. The level of phosphorylated PDGFRβ following PDGF-BB treatment was lower in TGFβ-stimulated than in non-stimulated ST2 cells and human MSCs (Figures 6A and 6B), indicating that PDGF stimulates PDGFRβ to a lesser extent in CAFs than in MSCs. These results suggest that the functions of PDGFRβ in MSCs are altered upon TGFβ stimulation (Figure 6C).

## DISCUSSION

This study demonstrates that PDGFRβ strongly interacts with TGFβR upon induction of the differentiation of MSCs into CAFs by TGFβ stimulation. Moreover, the level of phosphorylated PDGFRβ was lower in CAFs than in ST2 cells following PDGF treatment (Figures 6A and 6B), indicating that PDGF stimulates PDGFRβ to a lesser extent in CAFs than in MSCs. PDGFRs promote cell proliferation upon PDGF stimulation by activating the Ras, Erk, and MAPK pathways [12, 27] and also promote cell migration and differentiation by activating the PI3K pathway [27]. The response of PDGFRβ to PDGF stimulation was decreased in CAFs (Figures 6A and 6B), suggesting that the main function of PDGFRs in CAFs is to maintain these cells rather than to modulate their proliferation. This functional change may accelerate metastasis and malignancy in tumor microenvironments.

PDGFRα and PDGFRβ form homodimers and heterodimers upon ligand stimulation [12]. The functions of these isoforms reportedly differ [31]. Chemotaxis is induced by homodimeric and heterodimeric PDGFRβ, but not by homodimeric PDGFRα [31]. The expression levels of PDGFRα and PDGFRβ have been suggested to differ in several cell types following TGFβ stimulation; expression of PDGFRβ generally increases, while that of PDGFRα decreases [32, 33]. Moreover, bioinformatics screening of colorectal cancer patients demonstrated that expression of PDGFRβ is strongly correlated with that of TGFβ signaling-related genes [34]. We showed that TGFβ stimulation increased mRNA expression of PDGFRβ in ST2 cells and primary human MSCs, but decreased that of PDGFRα (Figures 3A and 3B). These findings imply that changes in the expression levels of PDGFRα and PDGFRβ alter cellular behaviors in some cell types and that PDGFRβ helps to transmit TGFβ signaling and to accelerate tumor malignancy.

Complex formation between PDGFRβ and TGFβR was increased in ST2 cells immediately after TGFβ stimulation (Figure 5A). Therefore, the functional changes in PDGFRβ may be due to its interaction with TGFβR and subsequent TGFβ signaling. Transmembrane receptors are mainly internalized via clathrin-mediated or caveolin-mediated endocytosis [35]. Signaling differs between these two types of internalization [36]. PDGFRβ endocytosed in a clathrin-mediated manner upon stimulation with a low concentration of PDGF promotes cell migration, whereas PDGFRβ endocytosed in a caveolin-mediated manner upon stimulation with a high concentration of PDGF accelerates cell proliferation [37]. Moreover, clathrin-mediated endocytosis of TGFβR induces Smad activation and cell differentiation [38]. Based on these results and our findings, we speculate that complex formation between PDGFRβ and TGFβR promotes clathrin-mediated endocytosis of both receptors in CAFs and that this promotes cell differentiation and migration.

Pre-treatment with tranilast, imatinib, and pirfenidone, which inhibit differentiation into CAFs, attenuated the increase in complex formation between PDGFRβ and TGFβR in ST2 cells upon TGFβ stimulation (Figure 5B). Tranilast was reported to suppress differentiation into CAFs by inhibiting protein synthesis of TGFβ [19]; however, the detailed underlying molecular mechanism is unclear. This drug also inhibits release of chemical mediators from mast cells by suppressing Ca^2+^ influx and stabilizing the cell membrane [39]. We speculate that tranilast inhibits TGFβ signaling by stabilizing the plasma membrane of MSCs and decreasing membrane plasticity and complex formation between PDGFRβ and TGFβR. Imatinib inhibits the proliferation of cancer cells by suppressing the kinase activities of c-ABL, BCR-ABL, and c-KIT [40]. Moreover, imatinib inhibits the activity PDGFRβ [41] and suppresses that of TEL-PDGFRβ, which is a fusion tyrosine kinase generated in chronic myeloproliferative diseases [42]. Therefore, we speculate that imatinib suppresses complex formation between PDGFRβ and TGFβR by changing the conformation of PDGFRβ or by competitively occupying the TGFβR-binding site of PDGFRβ. We suggest that new compounds/drugs that inhibit this interaction should be screened to suppress differentiation into CAFs.

The Smad2/3-mediated canonical and Smad2/3-independent non-canonical pathways are activated upon stimulation of cells with TGFβ [28, 29]. Thus, we hypothesized that PDGFRβ and TGFβ signaling cooperatively promote the activation of Smad2/3 during the differentiation of MSCs into CAFs; however, the level of phosphorylated Smad2/3 after TGFβ stimulation did not significantly differ between control and PDGFRβ-knockdown ST2 cells (data not shown). However, the levels of phosphorylated Akt and Erk, which contribute to the non-canonical TGFβ signaling pathways [29], were lower in PDGFRβ-knockdown ST2 cells than in control ST2 cells upon TGFβ stimulation (Figure 5E). Akt activation was suggested to promote the migration of CAFs derived from human breast tumor tissue by inducing phosphorylation of the actin-binding protein Girdin [43]. Functional suppression of GSK3β by Akt or Erk reportedly stabilizes the TGFβ target gene Snail and promotes epithelial-to-mesenchymal transition [44, 45]. Based on these findings, we speculate that non-canonical TGFβ signaling pathways control cell differentiation and migration by remodeling the actin cytoskeleton. The increase in mRNA expression of αSMA upon TGFβ stimulation was attenuated in ST2 cells treated with PI3K, MEK, and Src inhibitors (Figure 5D). Autophosphorylation of PDGFRβ upon PDGF stimulation activates PI3K, which promotes cell proliferation by phosphorylating Akt, mTOR, and S6K [46]. By contrast, TGFβ-induced activation of Akt increases αSMA expression in an mTOR-independent manner via a mechanism involving upregulation of serum response factor and myocardin, which are transcription factors that regulate the expression of genes encoding actin-binding proteins [47]. These findings indicate that the behaviors of signaling factors downstream of Akt differ between proliferating and differentiating cells. Taking our results together, PDGFRβ may activate non-canonical TGFβ signaling pathways and thereby promote cell differentiation and migration, rather than cell proliferation.

CAFs in tumor microenvironments play an important role in cancer malignancy. Therefore, an approach that targets CAFs in addition to cancer cells is required to prevent tumor progression, including cancer metastasis. Here, we revealed that PDGFRβ transmits TGFβ signaling by interacting with TGFβR during the differentiation of MSCs into CAFs and that the functions of PDGFRβ are altered in CAFs such that it promotes cell differentiation and migration rather than proliferation. Our finding that PDGFRβ is involved in TGFβ signaling will be useful for screening of compounds that inhibit differentiation into CAFs.

## MATERIALS AND METHODS

### Reagents

Recombinant TGFβ (Wako Pure Chemical, Tokyo, Japan) and recombinant PDGF-BB (Wako Pure Chemical) were dissolved in Milli Q water. Tranilast (Tokyo Kasei Kogyo Co., Tokyo, Japan), imatinib (Phoenix Pharmaceuticals, Belmont, CA, USA), pirfenidone (Tokyo Kasei Kogyo Co.), cromoglicate (Tokyo Kasei Kogyo Co.), bosutinib (KareBay Biochem, Ningbo, China), dasatinib (Cayman Chemical, Ann Arbor, MI, USA), CP673451 (AdooQ BioScience, Irvine, CA, USA), LY294002 (LC Laboratories, Woburn, MA, USA), PD98059 (Wako Pure Chemical), and PP2 (Cayman Chemical) were dissolved in dimethyl sulfoxide. The final concentration of dimethyl sulfoxide in each cell culture did not exceed 0.5% (v/v).

### Cell culture

ST2 and B16F1 cells were purchased from RIKEN Cell Bank (Tsukuba, Japan) and maintained in RPMI-1640 (Nacalai Tesque, Kyoto, Japan) supplemented with 10% fetal bovine serum (Life Technologies, Grand Island, NY, USA) and antibiotics (Nacalai Tesque) at 37°C in a humidified atmosphere of 5% CO_2_ in air. Human MSCs were purchased from Lonza Japan (Tokyo, Japan) and cultured in MSCGM™ BulletKit™ (Lonza Japan) according to the manufacturer’s instructions. ST2 cells were treated with TGFβ and PDGF-BB in culture medium supplemented with 1% fetal bovine serum for 24 h, and then the medium was replaced by fresh medium.

### siRNA transfection

ST2 cells were transiently transfected with PDGFRβ-targeting and control siRNAs (SIGMA, St. Louis, MO, USA) using Lipofectamine™ 2000 (Life Technologies) according to the manufacturer’s instructions. The sequences of the two oligonucleotide strands of the PDGFRβ-targeting siRNA duplex were as follows: sense, 5´-GCG AGA AGC AAG CCU UAA UTT-3´ and anti-sense, 5´-AUU AAG GCU UGC UUC UCG CTT-3´.

### RNA isolation and quantitative real-time PCR

Total RNA was isolated from ST2 cells using Sepasol-RNA I reagent (Nacalai Tesque) and reverse-transcribed using Rever Tra Ace^®^ qPCR RT Master mix (TOYOBO, Osaka, Japan). Alternatively, total RNA in the cell lysate was directly reverse-transcribed using a SuperPrep™ Cell Lysis & RT Kit for qPCR (TOYOBO). The resulting cDNA was mixed with THUNDERBIRD™ quantitative real-time PCR mix (TOYOBO) and subjected to quantitative real-time PCR using a LightCycler™ Nano Real-Time PCR System (Roche, Mannheim, Germany) and the following primers: mouse αSMA forward, 5´-TCC TCC CTG GAG AAG AGC TAC-3´ and reverse, 5´-TAT AGG TGG TTT CGT GGA TGC-3´; human αSMA forward, 5´-GAC AAT GGC TCT GGG CTC TG-3´ and reverse, 5´-TGC CAT GTT CTA TCG GGT AC-3´; mouse N-cadherin forward, 5´-AGC GCA GTC TTA CCG AAG G-3´ and reverse, 5´-GGC TCG CTG CTT TCA TAC TGA AC-3´; human N-cadherin forward, 5´-ACA GTG GCC ACC TAC AAA GG-3´ and reverse, 5´-CCG AGA TGG GGT TGA TAA TG-3´; mouse vimentin forward, 5´-CGG AAA GTG GAA TCC TTG C-3´ and reverse, 5´-CAC ATC GAT CTG GAC ATG CTG-3´; human vimentin forward, 5´-GAG AAC TTT GCC GTT GAA GC-3´ and reverse, 5´-GCT TCC TGT AGG TGG CAA TC-3´; TGFβ forward, 5´-CAA GGG CTA CCA TGC CAA C-3´ and reverse, 5´-AGG GCC AGG ACC TTG CTG-3´; mouse PDGFRα forward, 5´-TTA TCG AGT CAA TCA GCC CC-3´ and reverse, 5´-TTG AGC ATC TTC ACA GCC AC-3´; human PDGFRα forward, 5´-CCT GGT CTT AGG CTG TCT TC-3´ and reverse, 5´-GCC AGC TCA CTT CAC TCT CC-3´; mouse PDGFRβ forward, 5´-CCG GAA CAA ACA CAC CTT CT-3´ and reverse, 5´-TAT CCA TGT AGC CAC CGT CA-3´; human PDGFRβ forward, 5´-ACA CGG GAG AAT ACT TTT GC-3´ and reverse, 5´-GTT CCT CGG CAT CAT TAG GG-3´; and 18S ribosomal RNA forward, 5´-CGC CGC TAG AGG TGA AAT TC-3´ and reverse, 5´-TTG GCA AAT GCT TTC GCT C-3´. The reaction was performed at 95°C for 60 s, followed by 40 cycles at 95°C for 10 s and 60°C for 60 s. Relative mRNA expression was calculated after normalization against 18S ribosomal RNA expression. The sequences amplified by quantitative real-time PCR were inserted into the pDONR221 plasmid (Life Technologies) for absolute quantification of the cDNA copy number.

### Immunostaining

Cells were fixed in PBS containing 4% formaldehyde, permeabilized in PBS containing 0.1% Triton X-100, immunostained with a rabbit anti-αSMA primary antibody (Abcam, Cambridge, UK), and labeled with a secondary antibody conjugated with an Alexa Fluor dye (Life Technologies). Nuclei were stained with TO-PRO-3 iodide (Life Technologies). Fluorescence was detected using a Carl Zeiss LSM700 laser scanning confocal microscope (Prenzlauer, Berlin, Germany).

### Enzyme-linked immunosorbent assay (ELISA)

The TGFβ protein level in the culture medium was measured using the DuoSet ELISA Development System (R&D Systems Inc., Minneapolis, MN, USA) according to the manufacturer’s instructions.

### Transwell co-culture system

B16 and ST2 cells were seeded into the upper or lower chamber of a transwell culture plate (Falcon^®^, Corning Inc., Corning, NY, USA) for non-contact co-culture. To collect mRNA from B16 cells, ST2 cells were seeded into the upper chamber, treated with TGFβ for 24 h, and then co-cultured with B16 cells seeded into the lower chamber for 7 days. To evaluate invasion of B16 cells, ST2 cells were seeded into the lower chamber, treated with TGFβ for 24 h, and then co-cultured for 24 or 48 h with B16 cells seeded into the upper chamber, which was coated with Matrigel (Corning Inc). Non-invaded cells were removed using Accutase™ (Nacalai Tesque), and then B16 cells that had invaded through the Matrigel were fixed in PBS containing 4% formaldehyde and stained with crystal violet (Wako Pure Chemical) prepared in methanol.

### Immunoprecipitation

Cells were lysed on ice in lysis buffer, which comprised 20 mM Tris-HCl (pH 7.4), 150 mM NaCl, 5% glycerol, 1% NP-40, and a protease inhibitor cocktail (Roche). Samples were incubated overnight at 4°C with an anti-PDGFRβ antibody (Cell Signaling Technologies, Beverly, MA, USA) or control rabbit IgG (Wako Pure Chemical). Immunoprecipitated proteins were adsorbed onto immobilized protein G-coated magnetic beads (Merck Millipore, Berlin, Germany) and eluted with 0.1 M glycine-HCl (pH 3.0), which was subsequently neutralized with 0.5 M Tris-HCl (pH 8.0) containing 1.5 M NaCl.

### Western blotting

Cells were lysed on ice in lysis buffer, which comprised PBS (pH 7.4) containing 1% Triton X-100 and protease and phosphatase inhibitor cocktails (Roche). Identical amounts of protein from each sample and immunoprecipitate were separated by SDS-PAGE and transferred to PVDF membranes (Merck Millipore). The membranes were blocked and probed with primary antibodies specific for p-PDGFRβ, PDGFRβ, p-tyrosine, p-Akt, Akt, p-Erk1/2, Erk, and GAPDH (all from Cell Signaling Technologies) as well as TGFβR (Abcam). Immunolabeled proteins were detected using HRP-labeled secondary antibodies (Santa Cruz Biotechnology, Santa Cruz, CA, USA) and ECL Prime detection reagents (GE Healthcare, Buckinghamshire, UK). Signals were visualized using the ImageQuant LAS 4000 system (GE Healthcare).

### Statistical analysis

All data are expressed as the mean ± SD or the mean ± SE of at least three independent experiments, unless indicated otherwise. Statistical analyses were performed using the Student’s *t* test or an analysis of variance followed by the Bonferroni test, where applicable. A p value of < 0.05 was considered significant.

## Abbreviations

αSMA: α-smooth muscle actin
CAF: cancer-associated fibroblast
ELISA: enzyme-linked immunosorbent assay
MAPK: mitogen-activated protein kinase
MSC: mesenchymal stem cell
PDGF: platelet-derived growth factor
PDGFR: PDGF receptor
PI3K: phosphoinositide 3-kinase
TGFβ: transforming growth factor β
TGFβR: TGFβ receptor
TKI: tyrosine kinase inhibitor
